# A prospective study of maternal postnatal depressive symptoms with infant-feeding practices in a Chinese birth cohort

**DOI:** 10.1186/s12884-019-2559-1

**Published:** 2019-10-28

**Authors:** Tingting Sha, Xiao Gao, Cheng Chen, Ling Li, Gang Cheng, Xialing Wu, Qianling Tian, Fan Yang, Qiong He, Yan Yan

**Affiliations:** Department of Epidemiology and Medical Statistics, Xiangya School of Public Health, Central South University, Changsha, Hunan China

## Abstract

**Background:**

The first few weeks after childbirth are critical, as women may encounter lactation problems and postpartum depression during this period. However, it is still unclear whether early breastfeeding behaviours are related to the symptoms of postnatal depression (PND) in Chinese populations. Therefore, the current study aimed to investigate the association between symptoms of PND and infant feeding practices based on a large-scale Chinese cohort.

**Methods:**

A prospective study of the community-based cohort was conducted from January 2015 to December 2016. Infant feeding outcomes, including exclusive/partial breastfeeding and formula feeding, were assessed according to the WHO guidelines. Symptoms of PND were assessed by the Edinburgh Postnatal Depression Scale at 4 weeks postpartum. Multivariate generalized estimating equation models were applied to investigate the associations between depressive symptoms and infant feeding behaviours.

**Results:**

A total of 956 mother-infant pairs were included. Fifty-six mothers presented screen-positive symptoms of PND with a cut-off ≥10. The percentage of early breastfeeding initiation was 75.8%, while the average duration of exclusive breastfeeding was 3.90 ± 2.33 months. Postnatal depressive symptoms were associated with a shorter breastfeeding duration (8.02 vs. 6.32 months, *P* < 0.05) and earlier formula introduction (4.98 vs. 3.60 months, *P* < 0.05). After adjustments were made for covariates, postnatal depressive symptoms were associated with an increased risk of the discontinuation of exclusive and partial breastfeeding (*β* = − 0.049, *P* = 0.047 and *β* = − 0.082, *P* = 0.006, respectively). Compared to mothers without symptoms of PND, mothers with depressive symptoms were more likely to supplement formula for their infants in the first year of life (*β* =0.074, *P* = 0.016). These associations were still significant in the sensitivity analyses, using an EPDS cut-off of ≥13.

**Conclusions:**

Our findings indicate that depressive symptoms at 4 weeks postpartum are associated with the cessation of exclusive and partial breastfeeding duration and the introduction of formula in the 12 months of delivery. Early psychosocial assessment and social support should be offered to mothers in the early postpartum period to indirectly prevent adverse breastfeeding outcomes.

## Background

At present, an estimated 10–15% of women experience depressive symptoms during the postpartum period [1]. Postnatal depression (PND) is a severe mental disorder that is often characterized as a persistent low mood in new mothers [2]. The Diagnostic and Statistical Manual for Mental Disorders-Fifth Edition defines depression with peripartum onset as occurring during pregnancy or within the first 4 weeks postpartum [3]. Emerging research suggests that PND is associated with a series of adverse effects on obstetric and perinatal outcomes, such as infant behaviours, cognition or growth development, and maternal-infant interaction [4–7].

Breastfeeding is the optimal way to nourish and nurture babies; it is beneficial to both infants and mothers in the short and long term [8]. The World Health Organization (WHO) and many other authorities recommended that infants be exclusively breastfed for the first 6 months after delivery and should be continuously breastfed for at least 1 year [9]. However, only one-third of children globally are exclusively breastfed from 0 to 6 months, which is far from the recommendation [10, 11].

The low rate of exclusive breastfeeding during the first 6 months highlights the importance of exploring factors that might potentially affect breastfeeding practices. Depression in the early postpartum period is an important factor [12, 13]. However, research investigating the relationship between breastfeeding and postpartum depressive symptoms shows mixed results. Data from previous systematic reviews have indicated that PND was associated with early breastfeeding cessation, decreased breastfeeding duration, and increased breastfeeding difficulties [14, 15]. For instance, the result of a prospective study suggested that mothers with PND seem to promote the discontinuation of breastfeeding at 5 months postpartum [16]. Researchers also found that depressive symptoms at 1 week postpartum were associated with the discontinuation of breastfeeding at 4 and/or 8 weeks postpartum and an increased risk of breastfeeding problems [17]. Moreover, in a large prospective study of postpartum women, the authors found that depression at 2 weeks postpartum was associated with the cessation of breastfeeding at 12 weeks postpartum [18]. However, other prospective studies found no association between PND and breastfeeding [19–22]. A cohort study of 429 infants reported that PND was not associated with the interruption of exclusive breastfeeding at 2 months postpartum (RR =1.44; 95%CI: 0.68–3.06) [23]. Based on the cross-lagged panel design, a recent prospective study examined the bidirectional association between breastfeeding and postpartum depression and found no evidence of relationships between them [24].

The conflicting results may be primarily attributed to several methodological limitations [25] or variations in the measurements used to assess breastfeeding and perinatal symptoms [24]. Most studies investigating this association were secondary data analyses, cross-sectional designs or retrospective designs. Moreover, some studies did not continue to the end of the children’s first year of life. Additionally, any level of breastfeeding was considered rather than the categorization of breastfeeding as exclusive or partial [26].

In China, the prevalence of children with exclusive breastfeeding for the first 6 months is only 17% [27], and the duration of breastfeeding is insufficient [28]. Moreover, the available results suggested that a range of 4.70 to 43.12% of mothers in China experience PND [29, 30]. The first weeks after childbirth are critical, as women may experience lactation problems and postpartum depression during this period. However, it is still unclear how early breastfeeding experiences are related to postpartum depression in Chinese populations. Therefore, the aim of this study was to examine the associations between symptoms of PND during the first 4 weeks postpartum and infant feeding practices based on a prospective study of a longitudinal birth cohort in China.

## Methods

### Participants

This study was based on an in-progress community-based birth cohort study that is conducted in three community health service centres (CHSCs) in Changsha, a capital city of Hunan Province. Maternal and child health care records in Changsha are available in electronic form from the Community Health Management Information System (CHMIS) of Hunan Province. The baseline survey was performed at 1 month postpartum from 1 January 2015 to 31 December 2015, and the follow-up surveys were conducted during infants’ regular check-ups at the ages of 3 months, 6 months, 8 months and 12 months. Information was collected via face-to-face interviews conducted in the participants’ residences. The samples included in the final analysis were restricted to respondents who were permanent residents of Kaifu District, delivered live-born babies during the study period, provided complete healthcare records in the CHMIS, completed the assessment of PND, had no history of mental illnesses or brain diseases, agreed to participate in the study, and signed the written informed consents.

### Measures

Breastfeeding outcomes were assessed according to WHO-recommended definitions. Early breastfeeding initiation was defined as the provision of breast milk to infants within 1 h of giving birth, as recommended by the WHO [31]. Exclusive breastfeeding was defined as infants being fed with breast milk only, without any additional food or drink, not even water (allowable exceptions were expressed breast milk, oral rehydration solutions, and drops or syrups of vitamins, minerals, and medicines) [32]. Partial breastfeeding suggested that infants were fed breast milk regardless of whether they were provided with formula or complementary food. Formula feeding was defined as infants being fed with any formula. Complimentary food introduction was defined as infants being given solid or semi-solid food [33].

The Diagnostic and Statistical Manual for Mental Disorders-Fifth Edition defines postpartum depression as occurring within the first 4 weeks postpartum [3]. Maternal depressive symptoms were assessed at 4 weeks postpartum. The ten-item Edinburgh Postnatal Depression Scale (EPDS) is a simple and reliable screening instrument for the early detection of depression in the postnatal period, and this instrument has been extensively used worldwide. In this study, we assessed the symptoms of PND using the revised Chinese version of the EPDS, which has been demonstrated to have satisfactory psychometric properties [34]. Mothers reported on how often they experienced a symptom of depression in the past week on a four-point scale, with a total score ranging from 0 to 30. To identify and screen for probable postnatal depressive symptoms, a cut-off of ≥10 was used in this study. This cut-off value has been recommended as an optimum cut-off score when screening for clinical postpartum depression in Chinese mothers in Hong Kong [34] and mainland China [35, 36]. A cut-off of ≥10 has been also extensively used to screen for minor depression in English-speaking women [37, 38]. Moreover, we also performed sensitivity analyses using a cut-off of ≥13, which is usually recommended when screening for major postpartum depression [39]. Cronbach’s alpha coefficient for the EPDS was 0.86 in the present study.

### Data collection

The self-designed questionnaire, as well as maternal and child health care records in the CHMIS, were used to collect relevant information through face-to-face interviews conducted by trained investigators during infants’ regular check-ups. Daily infant feeding practices were collected by their mothers at 1, 3, 6, 8 and 12 months postpartum. Mothers were asked the following questions to measure infant feeding practices at each time point: 1) whether infants were fed breast milk; 2) whether infants were fed with any formula; 3) whether infants were fed with any complimentary food. Women were also asked to report the time of the cessation of breastfeeding, the introduction of formula, and the introduction of complementary food. Data on the early initiation of breastfeeding were collected by asking mothers the following question: “Did you initiated breastfeeding within one hour after delivery?”

Based on the previous literature [40], the following potential confounding factors that might affect infant feeding practices were identified and assessed: maternal age (< 25, 25–29, 30–34 or ≥ 35 years), maternal educational attainment (≤ junior school, senior school or ≥ college), household income per month (< 2000, 2000–5000, 5001–10,000 or > 10,000 yuan), pre-pregnancy BMI (< 18.5, 18.5–23.9, ≥ 24 kg/m^2^), passive and active smoking during pregnancy (yes or no), gestational week of birth (< 28, 28–36, 37–42 or > 42 week), mode of delivery (vaginal delivery or caesarean delivery), pregnancy-related complication (yes or no), using feeding bottle/nipple before breastfeeding (yes or no), parity (nulliparous or multiparous), infant gender (male or female) and other health-related factors. Maternal weight and height were measured by trained nurses at the CHSC within the first trimester as the proxies for pre-pregnancy weight and height. Pre-pregnancy BMI was calculated from pre-pregnancy weight (kg) divided by the square of height (m). Other measures such as multiple births, pregnancy-related complications, and gestational age were collected by extracting health records from the CHMIS. Information on maternal sociodemographic characteristics and other health-related factors were collected using self-designed questionnaires.

### Statistical analysis

Categorical variables were described as frequencies and percentages. Continuous variables were compared using two-sample t-tests or Wilcoxon’s signed-rank tests. Chi-square tests or Fisher’s exact tests were used to compare the categorical outcomes. Generalized estimating equation (GEE) models were used to compare the feeding outcomes based on the symptoms of PND (≥10). Sensitivity analyses were also performed to verify the associations between feeding outcomes and positive screening for major PND using a cut-off ≥13 on the EPDS. All statistical analyses were performed with SAS version 9.4 (SAS Institute, Inc., Cary, NC). Two-tailed *P* < 0.05 was considered statistically significant.

## Results

During the study period, a total of 1286 infants were born in the three CHSCs of the Kaifu District in Changsha. Among the study participants, 265 of the dyads were not permanent residents and had no healthcare records in the CHMIS, and 45 of the dyads refused to participate. Ultimately, 976 eligible mother-child dyads were included in the final birth cohort. Twenty of them had missing values on the EPDS. Thus, 956 of the dyads were included in the final analysis. Table 1 shows the baseline characteristics of the mother-child dyads. The average age of participants and the number of gestational weeks at delivery were 29.93 ± 3.45 years and 38.96 ± 1.51 weeks, respectively. The percentage of dyads with an early initiation of breastfeeding was 75.8%. The rates of mothers who screened positive for symptoms of PND (EPDS scores ≥10) and who screened positive for major PND (EPDS scores ≥13) were 5.9% (*n* = 56) and 3.0% (*n* = 29), respectively.

**Table 1:** Demographic characteristic of the sample (*N* = 956)

Variables	N	%
Maternal age (years)		
< 25	48	5.0
25–29	441	46.1
30–34	348	36.4
≥ 35	119	12.5
Maternal education attainment		
≤ Junior school	32	3.3
High school	122	12.8
≥ College graduate	802	83.9
Pre-pregnancy BMI^a^ (kg/m^2^)		
< 18.5	4	0.4
18.5–23.9	181	18.9
≥ 24	771	80.6
Household income (yuan)		
< 2000	31	3.2
2000–5000	521	54.5
5001–10,000	367	38.4
> 10,000	37	3.9
Mode of delivery		
Vaginal delivery	575	60.1
Cesarean delivery	381	39.9
Gestational age (weeks)		
< 28	0	0
28–36	46	4.8
37–42	910	95.2
> 42	0	0
Pregnancy-related complications		
No	819	85.7
Yes	137	14.3
Active smoking during pregnancy		
No	947	99.1
Yes	9	0.9
Passive smoking during pregnancy		
No	863	90.3
Yes	93	9.7
Parity		
Nulliparous	686	71.8
Multiparous	270	28.2
Screened positive for symptoms of PND (EPDS ≥10)		
No	900	94.1
Yes	56	5.9
Screened positive for major PND (EPDS ≥13)		
No	927	97.0
Yes	29	3.0
Using feeding-bottle/ nipple before breastfed		
No	791	82.7
Yes	165	17.3
Infant gender		
Male	493	51.6
Female	463	48.4
Initiation of breastfeeding (hours)		
< 1	725	75.8
1–24	118	12.3
> 24	113	11.8

^a^ *BMI* body mass index, *PND* postnatal depression

Table 2 shows the comparisons of infant feeding practices between mothers with and without depressive symptoms across time. Most of the participants with or without symptoms of PND breastfed their babies in the first month after delivery (89.3% vs. 97.4%). More than 70% of the participants without depressive symptoms breastfed their babies exclusively in the first month postpartum, while the exclusive breastfeeding rate of the mothers with depressive symptoms was 57.1% in the first month postpartum (*P =* 0.006). The partial breastfeeding rates of mothers with depressive symptoms were significantly lower than the rates of mothers with no depressive symptoms at 1, 3, 6, and 8 months postpartum, while this difference was not significant at 12 months postpartum. Compared to mothers without depressive symptoms, mothers with symptoms of PND were more likely to provide the supplementary formula for their infants in the first year of life.

**Table 2:** Comparisons of infant feeding practices between mothers with and without symptoms of PND ^a^ (EPDS ≥10) at each time point

Variables	1 month		3 months		6 months		8 months		12 months	
	% (n)^b^	*P*	% (n)	*P*	% (n)	*P*	% (n)	*P*	% (n)	*P*
Exclusive breastfeeding										
Overall	72.9 (697/956)		63.4 (600/946)		13.7 (128/932)					
with symptoms of PND	57.1 (32/56)	< 0.01	49.1 (27/55)	0.02	9.4 (5/53)	0.35	0	–	0	–
without symptoms of PND	73.9 (665/900)		64.3 (573/891)		14.0 (123/879)		0		0	
Partial breastfeeding										
Overall	97.0 (927/956)		89.7 (848/946)		76.1 (709/931)		62.8 (582/927)		29.1 (265/910)	
with symptoms of PND	89.3 (50/56)	< 0.01	74.5 (41/55)	< 0.01	56.6 (30/53)	< 0.01	47.2 (25/53)	0.02	23.1 (12/52)	0.32
without symptoms of PND	97.4 (877/900)		90.6 (807/891)		77.3 (679/878)		63.7 (557/874)		29.5 (253/858)	
Formula feeding										
Overall	27.1 (259/956)		36.3 (343/945)		55.6 (517/931)		68.6 (636/927)		85.2 (775/909)	
with symptoms of PND	42.9 (24/56)	< 0.01	28 (50.9)	0.02	69.8 (37/53)	0.03	84.9 (45/53)	0.01	92.3 (48/52)	0.14
without symptoms of PND	26.1 (235/900)		315 (35.4)		54.7 (480/878)		67.6 (591/874)		84.8 (727/857)	

^a^ *PND* postnatal depression

^b^ Data are expressed as percentages (numbers)

Table 3 presents the partial breastfeeding duration and the time of formula introduction between mothers with PND and mothers without depressive symptoms. The partial breastfeeding duration of the mothers with depressive symptoms was significantly shorter than the duration of the mothers without depressive symptoms (8.02 months vs. 6.32 months, *P* = 0.008). Compared to the mothers without symptoms of PND, mothers with symptoms of PND were more likely to introduce formula for their infants after giving birth (4.98 months vs. 3.60 months, *P =* 0.014).

**Table 3:** Comparisons of partial breastfeeding duration and formula introduction between mothers with and without symptoms of PND

Variables	Symptoms of PND ^a^ (EPDS ≥10), median (IQR)		*P*
	No (*n* = 900)	Yes (*n* = 56)	
Duration of partial breastfeeding (months)	8.02 (3.43)	6.32 (3.96)	0.008
The introduction of formula (months)	4.98 (3.93)	3.60 (3.58)	0.014

^a^ *PND* postnatal depression, *IQR* interquartile range

### Associations between symptoms of PND and partial/exclusive breastfeeding and formula feeding

The results of the GEE models of PND with infant feeding practices are summarized in Table 4. The data on active smoking among mothers were insufficient, so it was not included in the GEE model. After controlling for covariates such as maternal age, maternal education attainment, household income, and pre-pregnancy BMI, depressive symptoms at 4 weeks postpartum were associated with the cessation of exclusive (*β* = − 0.049; 95% CI: − 0.101, − 0.004; *P* = 0.047) and partial breastfeeding (*β* = − 0.082; 95% CI: − 0.140 -0.024; *P* = 0.006) at 12 months postpartum. Moreover, symptoms of PND were associated with the introduction of formula for infants in the first year of life (*β* = 0.074; 95% CI: 0.014, 0.134; *P* = 0.016). Women who initiated breastfeeding more than 24 h after giving birth were more likely to stop exclusive or partial breastfeeding (*β* = − 0.044 and − 0.035, respectively; *P* < 0.05 for both) than were women who breastfed their babies within 1 h. In comparison to pre-pregnancy BMI between 18.5 and 23.9 kg/m^2^, pre-pregnancy BMI less than 18.5 kg/m^2^ was associated with both partial (*β* = − 0.263) or exclusive breastfeeding cessation (*β* = − 0.198) and formula introduction (*β* =0.213). Higher childbearing age (older than 35 years old) was associated with the cessation of breastfeeding for infants (*β* =0.035). Additionally, compared to vaginal deliveries, caesarean deliveries were associated with a higher likelihood of formula introduction and the discontinuation of exclusive breastfeeding. No significant differences in exclusive or partial breastfeeding were found in the different maternal age groups. We also performed sensitivity analyses using the cut-off of ≥13, and the results can be found in Additional file 1: Table S1. Our findings suggested that the associations between major postpartum depression and infant feeding outcomes were still significant in the sensitivity analyses.

**Table 4:** Results of the generalized estimating equations of symptoms of PND with infant-feeding outcomes across time

Variables	Exclusive breastfeeding			Partial breastfeeding			Formula feeding		
	*β*	95% CI		*β*	95% CI		*β*	95% CI	
Maternal educational attainment									
≥ College graduate (ref)	–	–	–	–	–	–	–	–	–
≤ Junior school	−0.001	− 0.062	0.061	− 0.005	− 0.064	0.053	− 0.078	− 0.185	0.029
High school	− 0.002	− 0.032	0.029	− 0.016	− 0.046	0.013	0.015	− 0.032	0.061
Maternal age (years)									
25–29 (ref)	–	–	–	–	–	–	–	–	–
≤ 24	0.027	−0.018	0.071	0.036	−0.006	0.078	−0.022	−0.089	0.046
30–34	0.000	−0.024	0.024	0.001	−0.023	0.024	0.005	−0.029	0.038
≥ 35	0.020	−0.012	0.053	0.035*	0.005	0.065	−0.027	−0.075	0.022
Pre-pregnancy BMI (kg/m^2^)									
18.5–23.9 (ref)	–	–	–	–	–	–	–	–	–
< 18.5	−0.198*	−0.333	−0.064	− 0.263*	−0.394	− 0.133	0.213*	0.136	0.290
≥ 24	−0.006	−0.032	0.020	0.022	−0.004	0.047	−0.022	−0.058	0.013
Household income (yuan)									
5001–10,000 (ref)	–	–	–	–	–	–	–	–	–
≤ 2000	0.072	−0.008	0.152	−0.027	−0.091	0.037	0.072	−0.008	0.152
2001–5000	0.019	−0.012	0.050	−0.010	−0.031	0.011	0.019	−0.012	0.050
> 10,000	0.057	−0.020	0.135	−0.072	−0.147	0.003	0.057	−0.020	0.135
Initiation of breastfeeding (hours)									
< 1 (ref)	–	–	–	–	–	–	–	–	–
1–24	0.005	−0.028	0.038	0.010	−0.020	0.039	0.003	−0.043	0.048
≥ 24	−0.035*	−0.074	− 0.005	−0.044*	− 0.083	−0.004	0.037	−0.014	0.089
Delivery mode									
Vaginal delivery (ref)	–	–	–	–	–	–	–	–	–
Cesarean delivery	−0.043*	−0.066	− 0.021	−0.016	− 0.038	0.006	0.063*	0.031	0.094
Screen-positive symptoms of PND									
No (ref)	–	–	–	–	–	–	–	–	–
Yes	−0.049*	− 0.101	−0.004	− 0.082*	−0.140	− 0.024	0.074*	0.014	0.134
Gestational age (weeks)									
37–42 (ref)	–	–	–	–	–	–	–	–	–
28–36	−0.009	−0.055	0.038	−0.029	− 0.105	0.047	− 0.003	−0.052	0.047
Parity									
Nulliparous (ref)	–	–	–	–	–	–	–	–	–
Multiparous	−0.015	−0.039	0.010	−0.005	− 0.028	0.019	0.017	−0.018	0.052
Miscarriage									
No (ref)	–	–	–	–	–	–	–	–	–
Yes	0.010	−0.011	0.031	−0.017	− 0.038	0.005	− 0.006	−0.037	0.025
Pregnancy-related complications									
No (ref)	–	–	–	–	–	–	–	–	–
Yes	0.007	−0.024	0.039	0.000	−0.032	0.032	−0.009	−0.054	0.036
Passive smoking during pregnancy									
No (ref)	–	–	–	–	–	–	–	–	–
Yes	0.014	−0.021	0.049	0.013	−0.019	0.044	0.016	−0.031	0.064
Using bottle/ nipple before breastfeeding									
No (ref)	–	–	–	–	–	–	–	–	–
Yes	−0.009	− 0.040	0.023	− 0.004	−0.034	0.026	0.005	−0.039	0.048

^*^ *P* < 0.05

^a^ *BMI* body mass index, *CI* confidence interval, *PND* postnatal depression

## Discussion

This study provides insight into infant feeding practices among women who experience depressive symptoms during the early postpartum period in the context of China. Our results suggested that after adjustments were made for potential confounders, symptoms of PND at 4 weeks postpartum were associated with increased risks of the absence of best-practice infant feeding practices, including the higher chance of exclusive breastfeeding cessation at 6 months or partial breastfeeding cessation and formula introduction at 12 months postpartum. These results highlight the importance of screening for symptoms of PND as one of the possible factors of adverse feeding outcomes.

The prevalence of mothers with symptoms of PND in Changsha (5.9%) was found to be relatively low level in China (4.70%~ 43.12%) [29, 30]. One of the main reasons for the low PND rates in Changsha was the limited representativeness of the sample. The sample included in this study tended to be older, more educated, and of higher socioeconomic status compare to general mothers in China. Previous studies have suggested that these factors were protective factors preventing women from experiencing PND [41]. Additionally, in Chinese culture, given the inherent stigma of mental illness, concern for appearances may lead women to deny having PND and to refrain from seeking help from others [42], which might contribute to the low levels of depression in Changsha.

The present study showed that symptoms of PND were associated with the discontinuation of exclusive or partial breastfeeding. Consistent with our findings, previous studies demonstrated that mothers with PND were at higher risk of shorter full/partial breastfeeding duration [15, 43, 44]. McLearn et al. reported that mothers with depressive symptoms were less likely to continue breastfeeding through two to 4 months postpartum than were mothers without depressive symptoms [45]. Using an EPDS score > 10 as the cut-off point for PND, Galler et al. found that depressive symptoms at 7 weeks postpartum could predict breastfeeding practices at 7 weeks, 3 months, and 6 months postpartum. However, these associations became non-significant at 6 months postpartum [46]. Our findings also suggested that depressive symptoms in the early postpartum period may be associated with the early formula introduction. Using the same cut-off used in our study, Gagliardi et al. found that mothers with PND were more likely to bottle feed at 3 months and that the odds of bottle-feeding increased with EPDS scores [47]. Consistent with a previous study [48], we found that breastfeeding initiation was associated with infant feeding outcomes. The study also discovered that caesarean deliveries were related to the supplementation of formula and the discontinuation of breastfeeding for infants.

The following are possible explanations for the associations between the high levels of depression symptoms and poor feeding outcomes. Postpartum depression might cause negative effects on maternal self-esteem and cognition [49]. Moreover, women with depressive symptoms may have more inadequate interactions with their new-borns, such as less touching, sensitivity, and skin to skin, which in turn increases the risk of breastfeeding difficulties, lack of confidence in their ability to breastfeed their babies, and decreased satisfaction related to their infant feeding practices [50].

This study provides more evidence for the role of postpartum depressive symptoms in feeding behaviours, especially for the Chinese population. The EPDS is a simple and reliable screening instrument for the early detection of depression in the postnatal period. Based on this screening instrument, our results highlight that even small benefits are achievable in terms of preventing non-optimal feeding practices by reducing the incidence of postpartum depression. Targeted and appropriate interventions, such as psychosocial assessment and social support, both from health professionals and from family and friends, including women’s partners/husbands, should be provided for mothers with symptoms of PND, especially for women of low socioeconomic status, as they are at increased risk for depression.

### Strengths

This study has several strengths. This study used a prospective longitudinal birth cohort. Thus, the data for feeding practices were collected prospectively; this design helps evaluate the direction of the proposed relationship between symptoms of PND and infant feeding behaviours [2]. A number of potential covariates identified from the prior literature were included in multivariate models. Moreover, the GEE model is suitable for analysing the longitudinal data, which can minimize the accumulation of Type I error from multiple endpoint comparisons [51]. One of the strengths of GEE is that we do not need to identify the exact distribution of the various breastfeeding outcomes across time but rather only need to provide the mean structure for the GEE model to perform the analysis [52].

### Limitations

The study has some limitations. The sample included in the current study is rather homogeneous, as participants are predominantly from the city, are older and are well educated with a high average household income, representing a healthier segment of the population. Thus, the generalization of the results to the general population of childbearing-aged women in China should be exercised with caution. Although we had adjusted for a range of potential confounders of feeding practices, there are still many other relevant prenatal and perinatal factors that were not included in our analysis, such as prenatal depressive symptoms, adverse life events, social support, partner relationship, breastfeeding self-efficacy, and pre-birth intentions to breastfeed. These unmeasured factors may affect the actual associations between maternal symptoms of PND and feeding practices. Additionally, postpartum depressive symptomatology in this study was based on mothers’ self-report. Future studies should include diagnostic interviews to ascertain PND clinically. Finally, this study revealed that the symptoms of PND at 4 weeks postpartum were associated with infant feeding outcomes at 12 months postpartum, but these associations must be interpreted cautiously because they were generated at only one evaluation of the depressive symptoms at 4 weeks postpartum, which might not be enough to determine the apparent longitudinal association. Hence, a well-designed, large-scale cohort study, which can provide a clear operationalized definition for infant feeding outcomes and screened symptoms of PND, should be further conducted to explore the longitudinal association between symptoms of PND and breastfeeding practices.

## Conclusion

In summary, our findings suggest that among mothers, symptoms of PND at 4 weeks postpartum are associated with the non-optimal infant feeding practices in the 12 months of delivery in the context of China. These results support the importance of screening mothers with depressive symptomatology during the first few weeks after childbirth. Early psychosocial assessment and social support should be offered to mothers in the early postpartum period to indirectly prevent adverse breastfeeding outcomes.

## Supplementary information

**Additional file 1: Table S1.** Results of the generalized estimating equations of screen-positive for major PND ^a^ (cutoff ≥13) with infant-feeding outcomes across time

## Data Availability

The datasets used and/or analyzed during the current study are available from the corresponding author on reasonable request.

## Abbreviations

CHMIS: Community Health Management Information System
CHSCs: Community Health Service Centers
CI: Confidence interval
EPDS: Edinburgh Postnatal Depression Scale
GEE: Generalized estimating equation
IQR: Interquartile range
PND: Postnatal depression
WHO: World Health Organization
