# Supplementary material for: A prospective study of maternal postnatal depressive symptoms with infant-feeding practices in a Chinese birth cohort
Source: BMC Pregnancy Childbirth. 2019 Oct 28;19:388. doi: 10.1186/s12884-019-2559-1 (PMC6819524; doi:10.1186/s12884-019-2559-1)
Supplement: Supplementary file 1 — Additional file 1: Table S1. Results of the generalized estimating equations of screen-positive for major PND a (cutoff ≥13) with infant-feeding outcomes across time [file 12884_2019_2559_MOESM1_ESM.docx]

**Supplementary Table 1** Results of the generalized estimating equations of screen-positive for major PND ^a^ (cutoff ≥13) with infant-feeding outcomes across time

| Variables | Exclusive breastfeeding | | | Partial breastfeeding | | | Formula feeding | | |
| --- | --- | --- | --- | --- | --- | --- | --- | --- | --- |
|  | $\beta$ | 95% CI | | $\beta$ | 95% CI | | $\beta$ | 95% CI | |
| Maternal educational attainment | | |  |  |  |  |  |  |  |
| ≥College graduate (ref) | - | - | - | - | - | - | - | - | - |
| ≤Junior school | 0.002 | -0.060 | 0.063 | -0.002 | -0.060 | 0.056 | -0.081 | -0.188 | 0.026 |
| High school | -0.001 | -0.031 | 0.030 | -0.015 | -0.044 | 0.015 | 0.014 | -0.033 | 0.060 |
| Maternal age (years) | | |  |  |  |  |  |  |  |
| 25-29 (ref) | - | - | - | - | - | - | - | - | - |
| ≤24 | 0.025 | -0.020 | 0.069 | 0.034 | -0.008 | 0.075 | -0.019 | -0.087 | 0.048 |
| 30-34 | 0.000 | -0.024 | 0.024 | 0.000 | -0.023 | 0.024 | 0.005 | -0.029 | 0.039 |
| $\geq$35 | 0.021 | -0.011 | 0.054 | 0.036* | 0.006 | 0.066 | -0.028 | -0.076 | 0.020 |
| Pre-pregnancy BMI (kg/m^2^) | | |  |  |  |  |  |  |  |
| 18.5-23.9 (ref) | - | - | - | - | - | - | - | - | - |
| <18.5 | -0.198* | -0.333 | -0.063 | -0.263* | -0.395 | -0.132 | 0.213* | 0.135 | 0.290 |
| ≥24 | -0.006 | -0.032 | 0.020 | 0.021 | -0.005 | 0.047 | -0.022 | -0.057 | 0.014 |
| Household income (yuan) | | |  |  |  |  |  |  |  |
| 5001-10000 (ref) | - | - | - | - | - | - | - | - | - |
| $\leq$2000 | -0.046 | -0.107 | 0.015 | -0.028 | -0.091 | 0.036 | 0.072 | -0.007 | 0.151 |
| 2001-5000 | -0.021 | -0.042 | 0.001 | -0.011 | -0.031 | 0.010 | 0.020 | -0.011 | 0.051 |
| $>$10000 | -0.055 | -0.118 | 0.009 | -0.070 | -0.145 | 0.006 | 0.056 | -0.023 | 0.134 |
| Initiation of breastfeeding (hours) | | |  |  |  |  |  |  |  |
| $<$1 (ref) | - | - | - | - | - | - | - | - | - |
| 1-24 | 0.005 | -0.028 | 0.038 | 0.008 | -0.022 | 0.038 | 0.003 | -0.043 | 0.048 |
| ≥24 | -0.036* | -0.077 | -0.001 | -0.043* | -0.082 | -0.004 | 0.045 | -0.004 | 0.095 |
| Delivery mode | | |  |  |  |  |  |  |  |
| Vaginal delivery (ref) | - | - | - | - | - | - | - | - | - |
| Cesarean delivery | -0.044* | -0.067 | -0.022 | -0.014 | -0.036 | 0.008 | 0.064* | 0.033 | 0.094 |
| Screen-positive for major PND | |  |  |  |  |  |  |  |  |
| No (ref) | - | - | - | - | - | - | - | - | - |
| Yes | -0.054* | -0.130 | -0.022 | -0.084* | -0.166 | -0.003 | 0.077* | 0.003 | 0.156 |
| Gestational age (weeks) | | |  |  |  |  |  |  |  |
| 37-42 (ref) | - | - | - | - | - | - | - | - | - |
| 28-36 | -0.008 | -0.055 | 0.038 | -0.002 | -0.052 | 0.047 | -0.030 | -0.105 | 0.046 |
| Parity | | |  |  |  |  |  |  |  |
| Nulliparous (ref) | - | - | - | - | - | - | - | - | - |
| Multiparous | -0.018 | -0.042 | 0.007 | -0.005 | -0.027 | 0.018 | 0.019 | -0.016 | 0.054 |
| Miscarriage | | |  |  |  |  |  |  |  |
| No (ref) | - | - | - | - | - | - | - | - | - |
| Yes | 0.013 | -0.008 | 0.033 | -0.012 | -0.033 | 0.010 | -0.009 | -0.040 | 0.022 |
| Pregnancy-related complications | | |  |  |  |  |  |  |  |
| No (ref) | - | - | - | - | - | - | - | - | - |
| Yes | 0.009 | -0.022 | 0.039 | 0.001 | -0.030 | 0.033 | -0.005 | -0.048 | 0.038 |
| Passive smoking during pregnancy | | |  |  |  |  |  |  |  |
| No (ref) | - | - | - | - | - | - | - | - | - |
| Yes | 0.015 | -0.019 | 0.049 | 0.016 | -0.015 | 0.047 | 0.012 | -0.035 | 0.059 |
| Using bottle/ nipple before breastfeeding | | |  |  |  |  |  |  |  |
| No (ref) | - | - | - | - | - | - | - | - | - |
| Yes | -0.009 | -0.040 | 0.022 | -0.004 | -0.034 | 0.026 | 0.005 | -0.038 | 0.047 |

^*^ *P*<0.05.

^a^ *BMI* body mass index, *CI* confidence interval, *PND* postnatal depression.
